# Comparison of Poly (ADP-ribose) Polymerase Inhibitors (PARPis) as Maintenance Therapy for Platinum-Sensitive Ovarian Cancer: Systematic Review and Network Meta-Analysis

**DOI:** 10.3390/cancers12103026

**Published:** 2020-10-18

**Authors:** Amos Stemmer, Inbal Shafran, Salomon M. Stemmer, Daliah Tsoref

**Affiliations:** 1 Sackler Faculty of Medicine, Tel Aviv University, Tel Aviv 6997801, Israel; amos@mail.tau.ac.il; 2 Internal Medicine II, Cardiology, Medical University Vienna, General Hospital Vienna, AKH, 1090 Vienna, Austria; inbal.shafran@gmail.com; 3 Davidoff Center, Rabin Medical Center, Petah Tikva 4941492, Israel; daliahts@clalit.org.il

**Keywords:** adverse event, network meta-analysis, ovarian cancer, overall survival, poly (ADP-ribose) polymerase inhibitor, platinum-sensitive, progression-free survival

## Abstract

**Simple Summary:**

Poly (ADP-ribose) polymerase inhibitors (PARPis; inhibitors of a family of enzymes that are primary involved in DNA repair) are considered to be the drug of choice in maintenance therapy for platinum-sensitive ovarian cancer. However, despite the FDA approval of three such agents and their availability in clinical practice, thus far, no clinical trial investigated them in a head-to-head direct comparison. In this study, we used a statistical approach that allows comparing direct and indirect evidence (network meta-analysis) in order to compare the three FDA-approved PARPis (olaparib, niraparib and rucaparib). To this end, we used data from six randomized control trials involving a total of 2270 ovarian cancer patients. Interestingly, we found no significant differences in clinical outcomes (overall survival and progression-free survival) between the three agents. However, niraparib was found to be associated with higher risk of certain adverse events (thrombocytopenia, neutropenia, constipation, and headaches) compared to the other two PARPis.

**Abstract:**

Background: Three PARPis (olaparib, niraparib and rucaparib) are currently FDA-approved as maintenance therapy in newly diagnosed and recurrent ovarian cancer. However, thus far, no trial has compared the three approved PARPis in the overall population, in patients with *BRCA* mutations, or in those with wild-type *BRCA*. Methods: A frequentist network meta-analysis was used for indirect comparisons between the different PARPis with respect to progression free survival (PFS), overall survival (OS), and adverse events. Results: Overall, six randomized clinical trials involving 2,770 patients, were included in the analysis. Results from the indirect comparisons revealed no statistically significant differences between the three PARPis with respect to PFS or OS in the entire population and in patients with mutated and wild-type BRCA, separately. Niraparib showed a statistically significant increased risk for grade 3 and 4 thrombocytopenia (risk-difference [RD] from placebo: 0.3; 95% confidence interval [CI], 0.27‒0.34) and any grade neutropenia (RD from placebo: 0.22; 95% CI, 0.18‒0.25) as compared with the other PARPis. Conclusion: No statistically significant difference was found between the three PARPis with respect to PFS or OS (overall and in subpopulations by *BRCA* status). There is, however, a statistical difference in toxicity as niraparib is associated with a greater risk for thrombocytopenia and neutropenia.

## 1. Introduction

Ovarian cancer is one of the leading causes of cancer death in women and the most common gynecologic cancer worldwide [1]. It is estimated that in 2018, there were 184,799 ovarian cancer deaths and 295,414 new cases of ovarian cancer worldwide [1]. Approximately 95% of ovarian cancer patients receive first-line treatment with carboplatin plus paclitaxel [2], with many achieving a complete response (CR) [3]. However, more than 80% of patients with advanced ovarian cancer experience recurrence, which is generally incurable [4].

Poly(ADP-ribose) polymerase (PARP) is a family of enzymes that are primarily involved in detecting single-strand DNA breaks, and trigger a cascade of events leading to DNA repair [5]. In chemotherapy-treated cancer cells, PARPs are assumed to repair chemotherapy-induced DNA damage. Therefore, adding PARP inhibitors (PARPis) to the treatment regimen promotes cancer cell death [6]. This mechanism of action paired with the centrality of BRCA proteins in the process of DNA repair resulted in the presumption that PARPis were primarily of value in tumors with a *BRCA1 or BRCA2* mutations [7]. While this has since been proven to be the case, it has also become evident that PARPis have additional efficacy in non-mutated *BRCA* homologous recombination-deficient tumors [8]. The use of PARPis is most established in ovarian cancer; however, the efficacy of these therapies has also been demonstrated in other cancer types such as breast cancer, castrate resistant prostate cancer, and pancreatic cancer [9]. Olaparib was the first PARPi to be approved by the United States food and drug administration (FDA) in 2014, followed by rucaparib in 2016. Both were first approved for advanced ovarian cancer with *BRCA* mutations. Niraparib was later approved as well (in 2017) for recurrent ovarian cancer. Olaparib and niraparib were subsequently approved as first line maintenance treatment (in 2017 and 2018, respectively) [10,11,12,13]. While all three agents have the same benzamide core, which is crucial for PARP binding, they differ in the rest of their chemical structure conferring them with different size and flexibility [14]. Interestingly, to date, no study comparing the efficacy or safety profile of these three drugs has been reported.

Network meta-analysis (NMA) is an approach used for comparing direct and indirect evidence and as such facilitates the collection of evidence from multiple trials and the comparison of relative effectiveness of multiple interventions [15]. In this study, we used NMA to compare the three approved PARPis, with respect to progression-free survival (PFS), overall survival (OS), and adverse events (AEs) in patients with platinum-sensitive ovarian cancer.

## 2. Results

**Figure 1:**
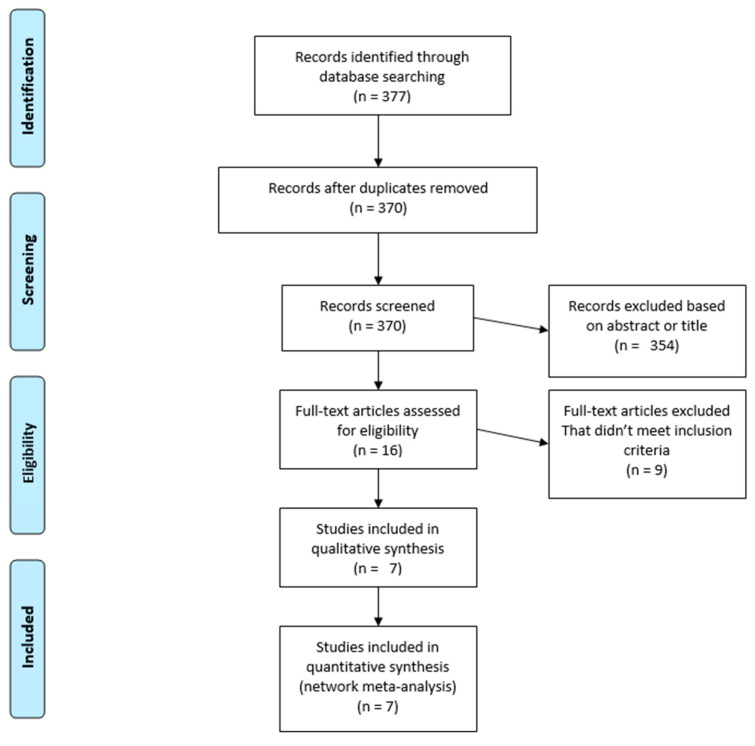
PRISMA flow chart of randomized control trials that are included and excluded.

During the search phase, a total of 370 publications were identified and screened. A total of 363 publications were excluded based on the title or abstract (*n* = 354) or the full-text article (*n* = 9). Thus, the NMA included data from six randomized clinical trials (RCTs) as reported in seven publications, that compared olaparib, niraparib or rucaparib to placebo. All these RCTs, combined, included a total of 2,270 patients (Figure 1).

### 2.1. Baseline Patient and Tumor Characteristics

Patient and tumor characteristics for all six RCTs included in the current analysis are presented in Table 1. The age of the patients ranged from 21 to 88 years. The median age in each of the six RCTs ranged from 53 to 62 years. The most common tumor location was the ovary (range in the study arms of the six RCTs, 79–87.5%), followed by fallopian tube (range, 2.2–16%) and peritoneum/others (range, 5–12.4%). Response to the most recent platinum-based chemotherapy ranged in the study arms of the six RCTs from 34% to 82% for CR and from 18% to 66% for partial response (PR). Four studies reported the number of prior chemotherapy regimens. Overall, 43–66% of patients in the study arms received 1–2 prior chemotherapy regimens and 34–57% received at least three prior chemotherapy regimens. All RCTs except for one [16] presented data regarding differentiation between *BRCA1* and *BRCA2* mutations. Three publications also presented data regarding homologous-recombination deficiency (HRD) rates [16,17,18]. However, only two of the three methods of describing HRD were consistent with each other and therefore results for the HRD group are not presented. Three RCTs reported disease stage [16,17,19]. In all three studies, the most common disease stage was stage 3 (range in the study arms, 64.2–72.9%). Ledermann et al. did not report the disease stage; however, the inclusion criteria entailed having high-grade serous features [20,21,22]. Similarly, Coleman et al. and Pujade-Lauraine et al. had an inclusion criteria of high-grade serous or endometrioid ovarian, primary peritoneal, or fallopian tube carcinoma [18,23].

All six RCTs included in the current analysis had a placebo arm. However, since other relevant information (e.g., PFS, OS, AEs) were not necessarily reported for all studies each NMA includes a different number of RCTs.

### 2.2. Study Description

Overall, of the six studies, three (Study 19, Solo1, and Solo2) compared olaparib to placebo, two studies (Nova and Prima) compared niraparib to placebo, and only 1 study (Ariel 3) compared rucaparib to placebo in this setting.

### 2.3. PFS and OS

**Figure 2:**
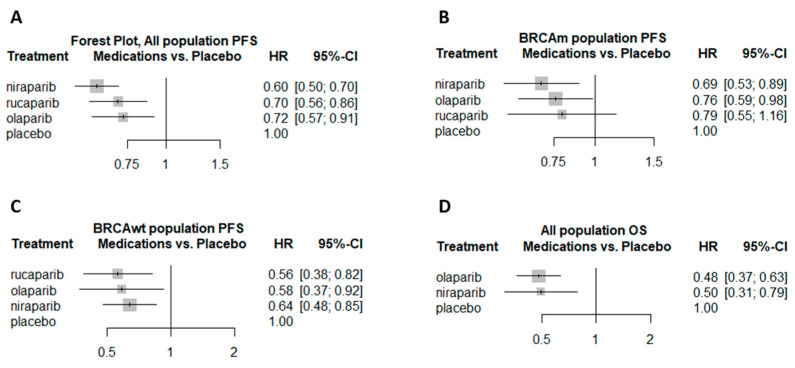
NMA forest plot of PFS and OS, comparing the different PARPis to placebo. Each subplot represents a different subgroup analysis. (**A**) All population PFS forest plot, (**B**) BRCAm population PFS forest plot, (**C**) BRCAwt population PFS forest plot, (**D**) All population OS forest plot

Overall, 5 trials presented PFS results for the overall population (patients with *BRCA1* or *BRCA2* mutations (BRCAm) and those with wild-type *BRCA* (BRCAwt) combined) [16,17,18,21,23]. All three PARPis demonstrated a statistically significant advantage over placebo. For niraparib, the hazard ratio [HR] relative to placebo was 0.6 (95% confidence interval (CI), 0.5 to 0.7); for rucaparib the HR was 0.7 (95% CI, 0.56 to 0.86); and for olaparib, the HR was 0.72 (95% CI, 0.57 to 0.91, Figure 2A, I^2^ = 5%).

Four trials presented PFS results for the BRCAm patient population [16,17,18,19,21]. Niraparib (HR 0.69; 95% CI, 0.53 to 0.89) and olaparib (HR 0.76; 95% CI, 0.59 to 0.98) both demonstrated a statistically significant advantage over placebo, whereas for rucaparib there was no statistical advantage compared to placebo (HR 0.79; 95% CI, 0.55 to 1.16, Figure 2B, I^2^ = 0%). Only three trials presented PFS results for the BRCAwt patient population [17,18,21]. Rucaparib (HR 0.56; 95% CI, 0.38 to 0.82), olaparib (HR 0.58; 95% CI, 0.37 to 0.92) and niraparib (HR 0.64; 95% CI, 0.48 to 0.85) all demonstrated a statistically significant advantage over placebo (Figure 2C).

Only two trials (an olaparib study and a niraparib study) presented OS results for the overall patient population [16,22]. Both agents demonstrated a statistically significant advantage over placebo (olaparib: HR 0.48; 95% CI, 0.37 to 0.63; niraparib: HR 0.5; 95% CI, 0.31 to 0.79) (Figure 2D). HRs with 95% CI as reported in the original trials are available in Table S1.

### 2.4. Adverse Events

All studies included in the analysis presented AE data. Compared to placebo, all three PARPis were associated with a statistically significant higher risk for anemia (any grade and only grade 3/4), as well as decreased appetite, dizziness, dyspnea, fatigue, nausea, neutropenia, and vomiting (any grade for all these AEs).

**Figure 3:**
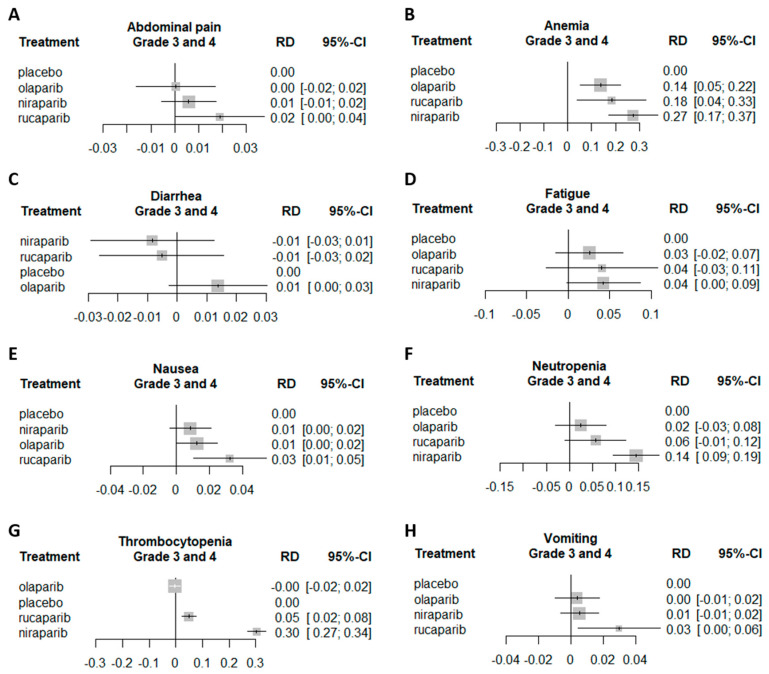
NMA forest plot of grade 3 and 4 AEs’ risk difference (RD) comparing the different PARPis to placebo. Each subplot represents a different AE. (**A**) Abdominal pain grade 3 and 4 forest plot, (**B**) Anemia grade 3 and 4 forest plot, (**C**) Diarrhea grade 3 and 4 forest plot, (**D**) Fatigue grade 3 and 4 forest plot, (**E**) Nausea grade 3 and 4 forest plot, (**F**) Neutropenia grade 3 and 4 forest plot, (**G**) Thrombocytopenia grade 3 and 4 forest plot, (**H**) Vomiting grade 3 and 4 forest plot.

**Figure 4:**
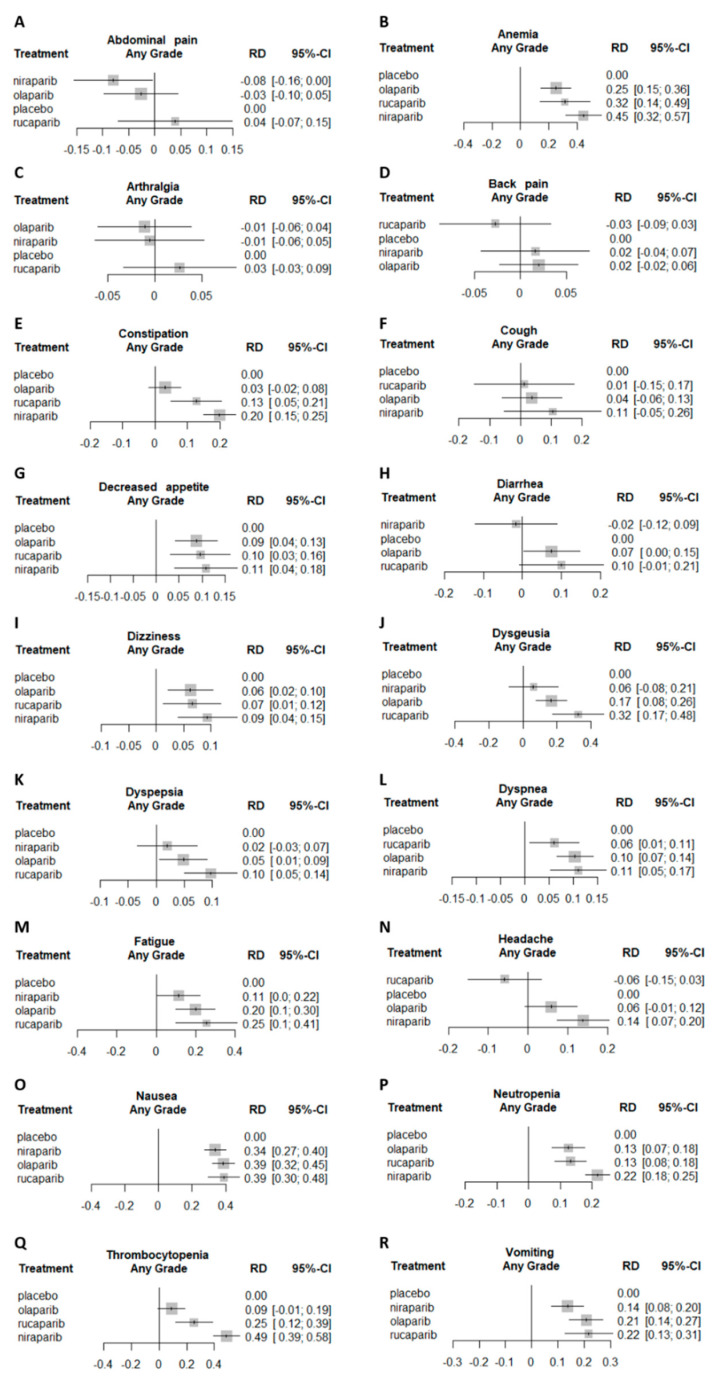
NMA forest plot of any grade AEs’ risk difference (RD) comparing the different PARPis to placebo. Each subplot represents a different AE. (**A**) any grade abdominal pain forest plot, (**B**) Any grade anemia forest plot, (**C**) Any grade arthralgia forest plot, (**D**) Any grade back pain forest plot, (**E**) Any grade constipation forest plot, (**F**) Any grade cough forest plot, (**G**) Any grade decreased appetite forest plot, (**H**) Any grade diarrhea forest plot, (**I**) Any grade dizziness forest plot, (**J**) Any grade dysgeusia forest plot, (**K**) Any grade dyspepsia forest plot, (**L**) Any grade dyspnea forest plot, (**M**) Any grade fatigue forest plot, (**N**) Any grade headache forest plot, (**O**) Any grade nausea forest plot, (**P**) Any grade neutropenia forest plot, (**Q**) Any grade thrombocytopenia forest plot, (**R**)Any grade vomiting forest plot.

The difference in AE risk between the study drug and placebo was assessed using risk difference (RD). For thrombocytopenia (any grade and only grade 3/4) RD was statistically significantly higher for niraparib than for olaparib and rucaparib. For neutropenia, this was also the case for any grade, but not for grade 3/4 only, where no statistical significance was observed between the PARPis. For any grade constipation (but not for only grade 3/4), the RD was statistically significantly higher for niraparib than for olaparib. For headache (grades 3/4), the RD was statistically significantly higher for niraparib than for rucaparib (Figure 3 and Figure 4). Additionally, for the following AEs, no increased RD was noted compared to placebo: Abdominal pain, back pain, arthralgia, and diarrhea. Furthermore, there were no statistically significant differences between the three PARPis in any grade anemia, arthralgia, back pain, cough, decreased appetite, diarrhea, dizziness, dysgeusia, dyspepsia, dyspnea, fatigue, nausea, and vomiting. AE as reported in the original trials are available in Table S2.

### 2.5. Risk of Biased Assessment

Overall, the risk of bias is low, as all trials supplied sufficient information about all five domains required by the updated Cochrane Collaboration’s Risk of Bias tool, namely, randomization process, deviation from intended intervention, missing outcome data, measurement of the outcome and selection of the reported result.

## 3. Discussion

Over the last decade, PARPis have become the standard maintenance therapy in platinum-sensitive recurrent ovarian cancer (regardless of *BRCA* status) [24]. Three PARPis are currently approved in this setting; however, no studies comparing them head-to-head are available. The current analysis, which is the first to compare PARPis in this setting, provides valuable information on their relative utility.

Our analysis demonstrated a similar efficacy for the three PARPis with respect to PFS and OS. However, with respect to AEs, statistically significant differences were observed, with niraparib being associated with a higher risk of thrombocytopenia and neutropenia compared to the other PARPis, more constipation (vs. olaparib) and more headaches (vs. rucaparib). Differences in toxicity profile are of clinical importance and could impact treatment decisions. It is important to note that both the Nova trial [17] and the Prima trial [16] initiated niraparib at a dose of 300 mg. However, the Nova trial protocol and treatment recommendations have since been amended due to data from a retrospective study that showed that a niraparib dose of 200 mg was effective and caused fewer AEs for patients with either a baseline body weight of less than 77 kg, a platelet count of less than 150,000 per cubic millimeter, or both [25]. Although this retrospective study appears to show that the AEs are dose dependent and that lowering the dose does not affect the PFS, this finding is yet to be confirmed in an RCT. It should be added that in all of the included RCTs, there were dose reductions due to AEs, ranging from 25.1% to 28% for olaparib, 54.6% for rucaparib and 66.5% to 70.9% for niraparib. Such dose reductions could have an effect on the efficacy and level of toxicity for each agent.

Differences between the three agents could be attributed to their selectivity as a recent study that profiled 10 clinical PARPis has shown that niraparib was more selective towards PARP1 and PARP2, whereas olaparib and rucaparib were more potent inhibitors of PARP1 but less selective [26]. Additional studies have concluded that rucaparib and olaparib have different in vitro affinity profile across a panel of diverse kinases. The analysis showed that rucaparib inhibited 9 of the 16 kinases tested, whereas olaparib did not inhibit a single one [27]. Additionally, a recent study using several computational methods followed by a comprehensive in vitro kinome screen revealed that these three agents differ in their binding affinity. Most notably, rucaparib was shown to inhibit CDK16, PIM3, and DYRK1B, whereas niraparib was shown to inhibit DYRK1A and DYRK1B [14]. The three agents differ in pharmacokinetics as well, niraparib has a mean half-life of 36h while olaparib and rucaparib mean half-life is 14 h and 17 h, respectively [28]. These findings could contribute to the observed differences in efficacy and toxicity between these agents.

Notably, some of the RCTs included in our analysis also reported on quality of life variables [29,30,31]; however, as these results were presented in each trial with different methods of assessment, these findings could not be included in the current NMA.

*BRCA1/2* status is hypothesized to have an effect on the AEs of PARPis [28]. As the RCTs included in the current study did not present the AE data stratified by *BRCA1/2* status, our analysis could not address this question.

Our study is limited by the small number of RCTs that were available for the analysis (as PARPis are relatively new therapeutics with a limited number of studies conducted thus far). To increase the sample size for our analysis, we included PARPis studies in both recurrent and newly diagnosed advanced ovarian cancer, since in both, patients were platinum-sensitive. Still, the number of patients did limit our ability to explore efficacy and safety in patient subpopulations. Another limitation of our study is the unavailability of OS data, as only two studies included in our NMA reported on OS. An additional limitation that is inherent to all the studies included in our NMA, and therefore to our study, involves the heterogeneity in the criteria for platinum-sensitive patients. All the studies, except for the Nova study, used the Response Evaluation Criteria in Solid Tumors (RECIST) version 1.0 or 1.1 criteria [32], or CA-125 levels for assessing those with CR or PR after platinum therapy. Lastly, some of the study participants underwent a surgical cytoreduction before the platinum therapy compromising the meaning of platinum sensitivity for such patients. The studies did not provide data on who underwent such surgery nor on the surgery outcomes, and therefore, this limitation could not be addressed in our analysis.

## 4. Methods

### 4.1. Systematic Review

We performed a systematic review according to the Preferred Reporting Items for Systematic Reviews and Meta-Analyses (PRISMA) guidelines and identified relevant literature from Embase, Pubmed, and the Cochrane Library. The search was not limited to any language, type, or dates. The search string used was: (“olaparib” AND “ovarian cancer” AND “maintenance” AND (“newly” OR “recurrent” OR “relapsed”)) OR (“niraparib” AND “ovarian cancer” AND “maintenance” AND (“newly” OR “recurrent” OR “relapsed”)) OR (“rucaparib” AND “ovarian cancer” AND “maintenance” AND (“newly” OR “recurrent” OR “relapsed”)) OR (“veliparib” AND “ovarian cancer” AND “maintenance” AND (“newly” OR “recurrent” OR “relapsed”)) OR (“PARP” AND “ovarian cancer” AND “maintenance” AND (“newly” OR “recurrent” OR “relapsed”)). One author examined all titles and abstracts. Full text of all relevant titles or abstracts was reviewed. To be included in the analysis, studies had to be phase 2 or 3 RCTs in platinum-sensitive recurrent or newly diagnosed ovarian cancer patients, who achieved CR or PR on platinum-based therapy, and who received either placebo or a PARPi as first-line maintenance therapy. In cases where trial updates were published, only the latest results were included in the NMA. A different author examined the final selected trials to ascertain they meet the inclusion criteria.

### 4.2. Outcomes of Interest

The primary outcome was PFS, calculated from the date of randomization to the date of progression (as defined by RECIST v1.1 criteria) or death. Secondary outcomes included OS, when available, which was defined as the time from randomization to death from any cause, and AEs.

### 4.3. Statistical Analysis

Hazard ratios (HR) values and 95% confidence intervals (CI) were extracted for PFS and OS when available. AEs were also retrieved and were categorized into grade 3 or 4 events and any grade events. RD and 95% CI from placebo were then calculated for each AE in each trial.

Mirza et al. 2016 [17] only reported HR and 95% CI for the PFS of patients with germline BRCA mutations and patients with no germline BRCA mutations. Subsequently, a meta-analysis was conducted only on those numbers in order to receive the HR and 95% CI for PFS for the overall patient population.

A frequentist random effects NMA was conducted on each of the outcomes. Results of the NMA are reported as HR with 95% CI for PFS and OS or RD with 95% CI for AEs. A Bayesian approach to NMA has been considered. However, recent studies show little to no difference in the results when comparing a Bayesian approach to a frequentist one [33,34]. AEs were included if they were reported by at least 4 trials, and if the events amounted to more than 1% of the patients included in the specific NMA. The analysis was performed in R version 3.6.2 [35], frequentist NMA was performed with the netmeta package. Heterogeneity of outcomes was assessed by I^2^. Internal validity of eligible studies was assessed according to the updated Cochrane Collaboration’s Risk of Bias tool [36].

## 5. Conclusions

Our analysis demonstrates no statistically significant differences between the three PARPis in any of the analyzed groups in terms of PFS or OS. The analysis did, however, demonstrate a difference in toxicity between the three agents, as niraparib was found to have a greater risk for thrombocytopenia and neutropenia. Additional OS results are pending from current studies, and would be useful in elucidating the relative roles of the different PARPis in platinum-sensitive ovarian cancer.

## Figures and Tables

**Table 1:** Patient/tumor characteristics by study and treatment.

Characteristic		Study 19 Ledermann 2012+ Friedlander 2018			Nova Mirza 2016		Ariel 3 Coleman 2017		Solo2 Pujade-lauraine 2017		Prima Gonzalez-martin 2019		Solo1 Moore 2018	
		Ola		Pla	Nir	Pla	Ruc	Pla	Ola	Pla	Nir	Pla	Ola	Pla
Median age (range) years		58 (21–89)		59 (33–84)	57,63 (33–84)	58, 61 (34–82)	61 (53–67)	62 (53–68)	56 (51–63)	56 (49–63)	62 (32–85)	62 (33–88)	53 (29–82)	53 (31–84)
Primary tumor location, *n* (%)	Ovary	119 (87.5)		109 (84.5)	314 (84.4)	149 (82.3)	312 (83)	149 (79)	164 (84)	86 (87)	388 (79.7)	201 (81.7)	220 (85)	113 (86)
	Fallopian	3 (2.2)		4 (3.1)	27 (7.2)	17 (9.4)	32 (9)	10 (5)	31 (16)	13 (13)	65 (13.3)	32 (13.0)	22 (8)	11 (8)
	Peritoneum	14 (10.3)		16 (12.4)	31 (8.3)	14 (7.8)	31 (8)	19 (10)	NR	NR	34 (7.0)	13 (5.3)	15 (6)	7 (5)
Time to progression after completion of penultimate platinum-based regime, *n* (%)	6 to 12 months	53 (39.0)		54 (41.9)	144 (38.7)	70 (38.7)	151 (40)	76 (40)	79 (40)	40 (40)	NR	NR	NR	NR
	above 12 months	83 (61.0)		75 (58.1)	228 (61.3)	111 (61.3)	224 (60)	113 (60)	117 (60)	59 (60)	NR	NR	NR	NR
Objective response to most recent platinum-based regimen, *n* (%)	Complete response	57 (41.9)		63 (48.8)	188 (50.5)	93 (51.4)	126 (34)	64 (34)	91 (46)	47 (47)	337 (69.2)	172 (70.0)	213 (82)	107 (82)
	Partial response	79 (58.1)		66 (51.2)	184 (49.5)	88 (48.6)	249 (66)	125 (66)	105 (54)	52 (53)	150 (30.8)	74 (30.0)	47 (18)	24 (18)
BRCA status, *n* (%)	BRCA1 or BRCA2	31 (22.8)		28 (21.7)	NR	NR	NR	NR	NR	NR	NR	NR	NR	NR
	BRCA1	25 (18.4)		20 (15.5)	85 (22.8)	43 (23.8)	80 (21)	37 (20)	132 (67)	61 (62)	NR	NR	191 (73)	91 (69)
	BRCA2	6 (4.4)		7 (5.4)	51 (13.7)	18 (9.9)	50 (13)	29 (15)	58 (30)	35 (35)	NR	NR	66 (25)	40 (31)
	Both	0		1 (0.8)	9 (2.4)	4 (2.2)	NR	NR	0	0	NR	NR	3 (1)	0
	Wildtype	98 (37)			NR	NR	245 (65)	123 (65)	NR	NR	NR	NR	NR	NR
	Mutation	111 (42)			NR	NR	130 (35)	66 (35)	NR	NR	NR	NR	NR	NR
Previous chemotherapy regimens, *n* (%)	1,2	59 (43)	63 (49)		226 (60.8)	107 (59.1)	231 (62)	124 (66)	110 (56) *	62 (63) *	NR	NR	NR	NR
	≥3	77 (57)	66 (51)		146 (39.2)	73 (40.3)	144 (38)	65 (34)	85 (44) *	37 (37) *	NR	NR	NR	NR
Stage, *n* (%)	1,2	NR	NR		45 (12.1)	15 (8.3)	NR	NR	NR	NR	0	0	0	0
	3	NR	NR		268 (72.0)	132 (72.9)	NR	NR	NR	NR	318 (65.3)	158 (64.2)	220 (85)	105 (80)
	4	NR	NR		58 (15.6)	33 (18.2)	NR	NR	NR	NR	169 (34.7)	88 (35.8)	40 (15)	26 (20)
ECOG, *n* (%)	0 1 2 Unknown	110 (80.9) 23 (16.9) 1 (0.7) 2 (1.5)	95 (73.6) 30(23.3) 2(1.6) 2(1.6)		251(67.4) 121(33.6) -- --	126(69.6) 55(30.4) -- --	280(75) 95 (25)	136 (72) 53 (28)	162 (83) 32 (16) 2 (1)	77 (78) 22 (22) 0	337 (69.2) 65 (26.3)	174 (70.4) 72 (29.3)	200 (76.9) 60 (23.1) 0	105 (80.2) 25 (19.1) 1 (0.8)

Abbreviations: Nir, Niraparib; NR, not reported; Ola, Olaparib; Pla, Placebo; Ruc, Rucaparib. * Only platinum-based.

## Supplementary Materials

The following are available online at https://www.mdpi.com/2072-6694/12/10/3026/s1, Table S1: PFS and OS by study and study population. Results are presented as HR (95% CI), Table S2: Adverse events in each study. Results are presented as adverse events number in arm (%).
