# Supplementary material for: Comparison of Poly (ADP-ribose) Polymerase Inhibitors (PARPis) as Maintenance Therapy for Platinum-Sensitive Ovarian Cancer: Systematic Review and Network Meta-Analysis
Source: Cancers (Basel). 2020 Oct 18;12(10):3026. doi: 10.3390/cancers12103026 (PMC7603267; doi:10.3390/cancers12103026)
Supplement: Supplementary file 1 [file cancers-12-03026-s001.pdf]

Comparison of Poly (ADP-ribose) Polymerase Inhibitors (PARPi's) as Maintenance Therapy for Platinum-sensitive Ovarian cancer: Systematic Review and Network Meta-analysis.

Amos Stemmer, Inbal Shafran, Salomon M. Stemmer and Daliah Tsoref

Table S1. PFS and OS by study and study population. Results are presented as HR (95% CI).

Population	Study 19 Ledermann 2012 + Friedlander 2018	Nova Mirza 2016	Ariel 3 Coleman 2017	Solo2 Pujade-Lauraine 2017	Prima Gonzalez-martin 2019	Solo1 Moore 2018
	Olaparib	Niraparib	Rucaparib	Olaparib	Niraparib	Olaparib
All population PFS	0.35 (0.25–0.49)	0.38 (0.3–0.49) *	0.36 (0.3–0.45)	0.3 (0.22–0.41)	0.62 (0.5–0.76)	NR
BRCAm PFS	0.18 (0.1–0.31)	0.27 (0.17–0.17)	0.23 (0.16–0.41)	NR	NR	0.3 (0.23–0.41)
BRCAwt PFS	0.54 (0.34–0.85)	0.45 (0.34–0.61)	0.58 (0.4–0.85)	NR	NR	NR
All population OS	0.73 (0.55–0.95)	NR	NR	NR	0.7 (0.44–1.11)	NR

Abbreviations: NR, not reported; OS, overall survival; PFS, progression free survival. * Calculated as described in the methods.

Table S2. Adverse events in each study. Results are presented as adverse events number in arm (%).

Adverse Event	Study 19 Ledermann 2012 + Friedlander 2018	Nova Mirza 2016	Ariel 3 Coleman 2017	Solo2 Pujade-Lauraine 2017	Prima Gonzalez-Martin 2019	Solo1 Moore 2018
	Olaparib $n = 136$	Niraparib $n = 367$	Rucaparib $n = 372$	Olaparib $n = 195$	Niraparib $n = 484$	Olaparib $n = 260$
Nausea grades 3 and 4	3 (0.2%)	11 (2%)	14 (3.76%)	5 (2.5%)	6 (1.2%)	2 (0.77%)
Nausea all grades	93 (68%)	270 (73.5%)	280 (75.2%)	148 (75.9%)	278 (57.4%)	201 (77.3%)
Fatigue grades 3 and 4	9 (0.66%)	30 (8.1%)	25 (6.7%)	8 (4.1%)	9 (1.8%)	10 (3.8%)
Fatigue all grades	66 (48.5%)	218 (59.4%)	258 (69.3%)	128 (65.6%)	168 (34.7%)	165 (63.4%)
Vomit grade 3 and 4	3 (2.2%)	7 (1.9%)	15 (4%)	5 (2.56%)	4 (0.8%)	1 (0.38%)
Vomit all grades	43 (31.6)	126 (34%)	136 (36.5%)	73 (37.4%)	108 (22.3%)	104 (40%)
Diarrhea grade 3 and 4	3 (2.2%)	1 (0.27%)	2 (0.5%)	2 (1%)	-	8 (3%)
Diarrhea all grades	31 (22.8%)	70 (19%)	118 (31.7%)	64 (32.8%)	-	89 (34.2%)
Decreased appetite all grades	25 (18.3%)	93 (25.3%)	87 (23.3%)	43 (22%)	-	51 (19.6%)
Abdominal pain grade 3 and 4	2 (1.4%)	4 (1.1%)	9 (2.4%)	5 (2.5%)	7 (1.4%)	4 (1.5%)
Abdominal pain all grades	24 (17.64%)	83 (22.6%)	111 (29.8%)	47 (24.1%)	106 (21.9%)	64 (24.6%)
Anemia grade 3 and 4	7 (5.1%)	93 (25.3%)	70 (18.8%)	38 (19.4%)	150 (31%)	56 (21.5%)
Anemia all grades	23 (16.9%)	184 (50.1%)	139 (37.3%)	85 (43.6%)	307 (63.4%)	101 (38.8%)
Dyspepsia all grades	22 (16.1%)	42 (11.4%)	54 (14.5%)	22 (11.2%)	-	43 (16.5%)
Dysgeusia all grades	19 (13.9%)	37 (10%)	146 (39.2%)	52 (26.6%)	-	68 (26.1%)

Cough all grades	18 (13.2%)	55 (14.9%)	54 (14.5%)	33 (16.9%)	-	42 (16.1%)
Arthralgia all grade	16 (11.7%)	43 (11.7%)	57 (15.3%)	29 (14.8%)	-	66 (25.3%)
Constipation all grades	17 (12.5%)	146 (39.7%)	136 (36.5%)	40 (20.5%)	189 (39%)	72 (27.6%)
Dyspnea all grades		71 (19.3%)	50 (13.4%)	23 (11.7%)	-	39 (15%)
Dizziness all grades	17 (12.5%)	61 (16.6%)	54 (14.5%)	26 (13.3%)	-	51 (19.6%)
Back pain all grades	16 (11.7%)	49 (13.3%)	45 (12%)	22 (11.2%)	-	40 (15.3%)
Headache all grades	25 (18.3%)	95 (25.8%)	37 (9.9%)	49 (25.1%)	126 (26%)	59 (22.6%)
Neutropenia grades 3 and 4	-	72 (19.6%)	25 (6.7%)	10 (5.1%)	62 (12.8%)	22 (8.4%)
Neutropenia all grades	-	111 (30.2%)	67 (18%)	38 (19.4%)	128 (26.4%)	60 (23%)
Thrombocytopenia grades 3 and 4	-	124 (33.7%)	19 (5.1%)	2 (1%)	139 (28.7%)	2 (0.7%)
Thrombocytopenia all grades	-	225 (61.3%)	104 (27.9%)	27 (13.8%)	222 (45.8%)	29 (11.1%)

Abbreviations: NR, not reported; OS, overall survival; PFS, progression free survival. * Calculated as described in the methods.

© 2020 by the authors. Licensee MDPI, Basel, Switzerland. This article is an open access article distributed under the terms and conditions of the Creative Commons Attribution (CC BY) license (http://creativecommons.org/licenses/by/4.0/).
